# Three-Dimensional, Tomographic Super-Resolution Fluorescence Imaging of Serially Sectioned Thick Samples

**DOI:** 10.1371/journal.pone.0038098

**Published:** 2012-05-25

**Authors:** Siddharth Nanguneri, Benjamin Flottmann, Heinz Horstmann, Mike Heilemann, Thomas Kuner

**Affiliations:** 1 Institute of Anatomy and Cell Biology, Heidelberg University, Heidelberg, Germany; 2 Bioquant Centre, University of Heidelberg, Heidelberg, Germany; 3 Biotechnology & Biophysics, Julius-Maximilians-University Würzburg, Würzburg, Germany; Julius-Maximilians-University Würzburg, Germany

## Abstract

Three-dimensional fluorescence imaging of thick tissue samples with near-molecular resolution remains a fundamental challenge in the life sciences. To tackle this, we developed *tomo*STORM, an approach combining single-molecule localization-based super-resolution microscopy with array tomography of structurally intact brain tissue. Consecutive sections organized in a ribbon were serially imaged with a lateral resolution of 28 nm and an axial resolution of 40 nm in tissue volumes of up to 50 µm×50 µm×2.5 µm. Using targeted expression of membrane bound (m)GFP and immunohistochemistry at the calyx of Held, a model synapse for central glutamatergic neurotransmission, we delineated the course of the membrane and fine-structure of mitochondria. This method allows multiplexed super-resolution imaging in large tissue volumes with a resolution three orders of magnitude better than confocal microscopy.

## Introduction

The spatial organization of subcellular components and proteins at the nanometer scale on the level of whole cells or tissues is challenging for modern light microscopy methods. Well-established light microscopy techniques are capable of imaging large volumes, yet limited in their spatial resolution to about 200 nm in the imaging plane and >500 nm along the optical axis. A superior axial resolution was demonstrated through ‘array tomography’, a technique which starts off with the preparation of ultrathin sections of tissue that are labeled via immunofluorescence and imaged using widefield or confocal microscopy [1]. This technique allowed imaging of thick samples with an axial resolution of 50 nm, determined by the thickness of the sections. Super-resolution fluorescence microscopy techniques can reach near-molecular spatial resolution down to tens of nanometers [2], [3] and several concepts with superior lateral and axial resolution have been demonstrated [3], [4], [5], [6]. However, reaching an almost isotropic resolution in 3D covering large volumes with a thickness of several micrometers is still demanding, yet necessary to study proteins in their native environment on the scale of entire cellular compartments, cells and tissues. A proof of principle using serial sectioning and STED microscopy attained a lateral resolution of 80 nm and an axial resolution of 70 nm, covering a volume of 3 µm×3 µm×1 µm [7]. _ENREF_8

In the current study, we investigated the calyx of Held, a sample that illustrates particularly well the requirements of imaging methods in terms of imaging volume and resolution. The calyx of Held is a glutamatergic presynaptic terminal located in the auditory pathway [8], [9]. This axo-somatic giant terminal engulfs a spherical cell of ∼20 µm diameter and contains 300–600 active zones for neurotransmitter release [10], [11], [12]. The calyx of Held can be considered the best studied glutamatergic synapse in the mammalian brain as it offers unique opportunities to link structure to function. Key questions regarding the nano-geometry of protein localization within active zones await 3D super-resolution microscopy to be addressed. Nano-geometry implies to locate several presynaptic proteins at once within a small area such as the active zone, a specialized compartment with a diameter of a few hundred nanometers. Knowing the exact positioning and copy number of proteins at the active zone will provide new insights into the mechanisms of synaptic transmission. Furthermore, it is essential to locate the plasma membrane and other functionally relevant organelles such as mitochondria in the context of active zone nano-geometry.

Here, we present an approach that combines array tomography and single-molecule localization-based super-resolution imaging with photoswitchable organic fluorophores following the protocol for direct stochastic optical reconstruction microscopy (*d*STORM) [13], which we termed *tomo*STORM. 2D *d*STORM imaging provides a lateral spatial resolution of ∼28 nm at an image size of up to 50×50 µm^2^. Array tomography provides an axial resolution of 40 nm or even less by virtue of the section thickness. By imaging many consecutive sections, *tomo*STORM can cover large 3D volumes spanning tens of micrometers in all directions.

**Figure 1:**
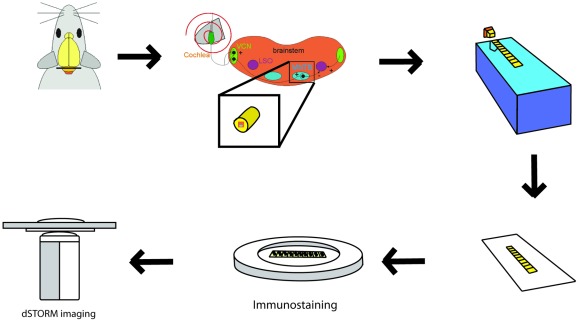
Schematic drawing of the experimental approach (see main text for further details).

Super-resolution microscopy at near molecular resolution must consider the extent of tissue preservation. Because it is known that LR-White embedding results in poor tissue preservation [14], the established array tomography approach [1] may not be appropriate to obtain meaningful protein localization data on the nanometer scale. Hence, fixation procedures and embedding resins conferring better tissue preservation need to be explored for their suitability for super-resolution imaging. Hydrophilic resins such as Lowicryl K4M, LR Gold and glycol methacrylate have been tested, with the latter providing the best compromise in maintaining genetically encoded fluorescence, reliably sectioning tissue at 70 nm thickness and ultrastructural preservation sufficient to resolve synaptic vesicles [15]. Here, we combine transcardial perfusion with PFA and embedding with the hydrophilic Lowicryl HM20 resin to obtain serial sections with a thickness below 50 nm and good ultrastructural preservation.

In summary, we introduce *tomo*STORM, a method that can sample multiple labels in large tissue volumes with a resolution of 28 nm×28 nm×40 nm.

## Results

**Figure 2:**
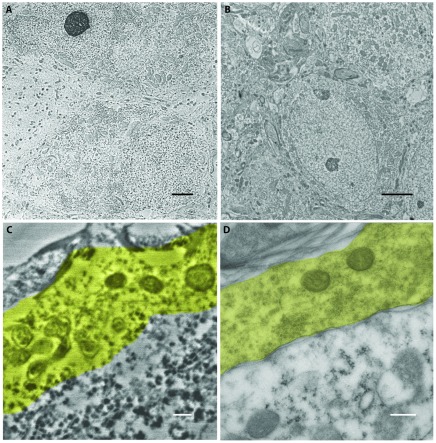
Comparison of electron micrographs of LRWhite embedded tissue of the auditory brain stem (A, C) and HM 20 embedded brain tissue (B, D). The high pressure-frozen HM 20 sections show much better ultrastructure preservation compared to LRWhite embedded sections. Scalebars in A and B 2 µm, in C and D 250 nm.

The experimental approach of *tomo*STORM starts with the preparation of serial sections that are cut from PFA-fixed tissue embedded in a polymer resin. These ribbon-like structures are transferred onto a glass substrate, immunolabeled and prepared for super-resolution imaging (Fig. 1). Because structural conservation is crucial when examining tissue at near molecular resolution, different embedding media were first investigated using electron microscopy. Being aware of the suboptimal tissue preservation in LRWhite [14], we nevertheless first revisited LRWhite embedding and subsequently tested Lowicryl HM20. Both resins support immunohistochemical staining and preparation of ultrathin sections below ∼50 nm thickness. We verified the level of structural conservation by electron microscopy of PFA-fixed nervous tissue. Auditory brainstem Tissue embedded with LRWhite and cut at 70 nm was largely disrupted (grainy regions and poor membrane structures, lacunar areas in Fig. 2A). A more detailed view confirms that membranes are poorly visible and that the cytoplasm appeared to be clustered (Fig. 2C). This was consistently found in numerous attempts of tissue preparation. In contrast, neuronal tissue embedded with HM20 and cut at 40 nm revealed a well organized tissue structure (Fig. 2B) and showed all structural details expected to be present (Fig. 2D) [16]. Thus, HM20 embedding results in tissue preservation superior to LRWhite and allows thinner sections to be prepared (even <35 nm), both crucial preconditions for fluorescence imaging of tissue at near molecular resolution.

**Figure 3:**
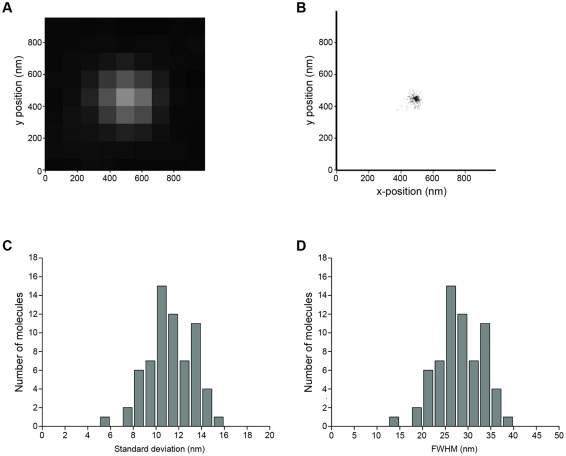
Determination of the spatial resolution. (A) point-spread function of a single fluorophore, (B) localization pattern of one single fluorophore that was localized multiple times through reversible photoswitching, (C) histogram of the standard deviation of localizations of 66 single-molecule point-spread functions (average standard deviation 12 nm) and (D) histogram of the full-width half-maximum (FWHM) of 66 single-molecule point-spread functions (average FWHM 28 nm).

Ribbons containing 10 to 50 consecutive sections cut at a thickness of 40 nm from rat brain stem were labeled with antibodies conjugated to the photoswitchable fluorophore Alexa Fluor 647 [17]. 2D super-resolution imaging was performed using the *d*STORM approach [13]. The localization accuracy was determined from single fluorophores that were localized multiple times (Fig. 3A, B). Each point-spread function recorded for a single fluorophore (Fig. 3A) was approximated by a Gaussian function, which next to the location of the single fluorophore provided a standard deviation of the localization of ∼12 nm (Fig. 3C) and a full-width half-maximum of ∼28 nm (Fig. 3D). The latter value can be interpreted as an approximate distance at which two single fluorophores can still be discriminated, and we refer to this value as lateral spatial resolution.

**Figure 4:**
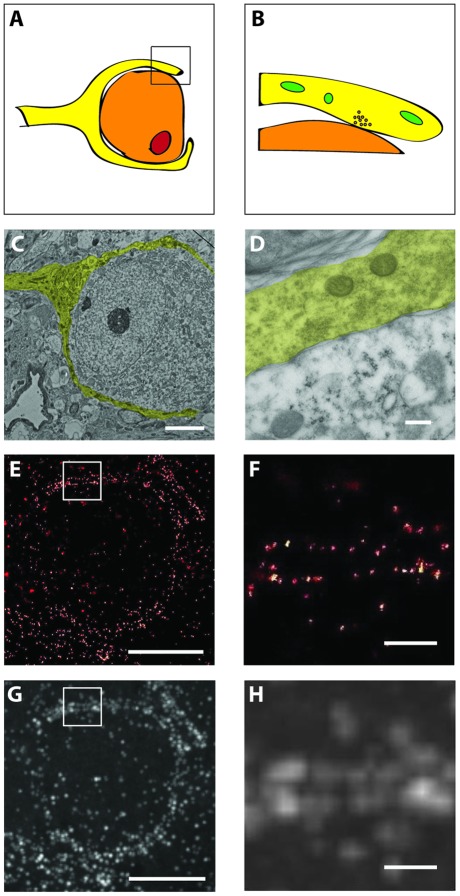
Structure of the calyx of Held on an overview scale (left panels) and on the scale of synaptic contacts (right panel). (A, B) Schematic representation, calyx (yellow), principal cell (orange), nucleus (orange). (C, D) Electron micrograph of the calyx of Held (yellow). (e, f) dSTORM images of the calyx of Held. (G, H) Widefield images of the calyx of Held. Scale bars 5 µm (left panels), 250 nm (right panels).

In a first experiment, we imaged tissue of the auditory brain stem from a rat expressing membrane bound GFP (mGFP) in the calyx of Held [10]. We chose mGFP because it is expected to reveal the course of the plasma membrane, a structure that can be easily related to known electron microscopy images of the calyx (Fig. 4). The structure of the calyx and its postsynaptic principal cell, with a diameter of approximately 20 µm, is illustrated in Figure 4A. The small box in is shown magnified to a typical electron microscopy ‘perspective’ in Figure 4B (synaptic vesicles and mitochondria lined out). These schematic views are further illustrated for the electron microscopic (Fig. 4C, D), *d*STORM imaging of antibody-labeled mGFP (Fig. 4E, F) and wide-field light microscopy ‘perspectives’. This comparison shows that the course of the membrane is adequately reproduced by imaging mGFP with *d*STORM (compare Fig. 4D and F, both at the same resolution).

**Figure 5:**
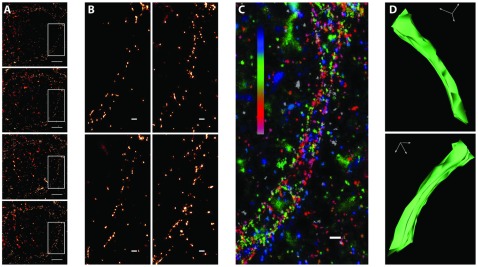
mGFP outlining the calyx membrane. (A) Four consecutive sections from a dataset of 53 sections containing segments of an entire calyx. Scale bars 2 µm. (B) Magnified views of the boxes illustrated in (A). Scale bars 250 nm. (C) Superposition of 15 consecutive aligned sections. Z-position of each section is color coded (top: blue, bottom: velvet). Scale bar 250 nm. (D) Two 3D views of the reconstructed segment shown in (C).

A ribbon of 53 serial sections cut at a thickness of 40 nm was labeled with an anti-GFP primary antibody and a secondary antibody coupled to Alexa Fluor 647. Imaging with *d*STORM revealed consecutive sections showing continuous variations of a typical calyx structure (Fig. 5A). The inset at larger magnification allows tracing the calyx membrane in each of the images, yet every section shows a different pattern of mGFP distribution. Noticeably and in contrast to confocal microscopy [10], the super-resolution images revealed a pronounced discontinuity of membrane labeling (Fig. 5A, B). This may reflect that mGFP expressed at micromolar concentrations can only label an incomplete fraction of the available membrane surface or that the labeling efficiency of the mGFP molecules present at the membrane is inefficient. Irrespectively, *d*STORM imaging achieves a resolution which approaches the size of a biomolecule allowing single sites of mGFP expression to be distinguished. Superposition of 15 sections spanning an axial distance of 600 nm generated a continuous representation of the plasma membrane (Fig. 5C, D). To demonstrate that *tomo*STORM is capable of imaging thick samples in 3D, we recorded super-resolution images of 53 consecutive thin sections of a calyx of Held segment (movie S1). In total, this image stack covers a volume of 40×40×2 µm^3^ with a resolution of 28 nm×28 nm×40 nm. A similar experiment using LRWhite as a resin did not reveal a 3D correlation of the 2D fluorescence patterns (not shown), consistent with the disrupted ultrastructure described above. Hence, we conclude that the super-resolution images obtained from HM20-embedded tissue closely reflect the known tissue ultrastructure.

**Figure 6:**
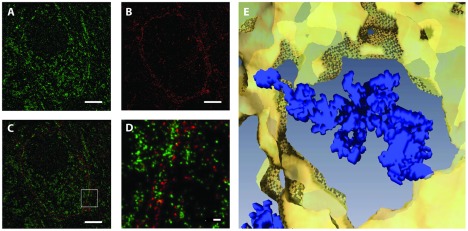
Dual color *d*STORM images of mitochondria and their localization within the calyx of Held. (A) Anti-cytochrome c oxidase stain. Nucleus spared. The soma of the principal neuron is densely populated with mitochodria. (B) Anti-mGFP stain of a single section through the calyx of Held. (C) Overlay of (A) and (B). (D) 3D rendering of a mitochondrion (blue) and surrounding membrane (yellow). Scale bars are 250 nm.

To identify the positioning of multiple structures in a single tissue volume, we modified the multi-step labeling approach used in the original array tomography approach [1]. Sections of mGFP-expressing calyces were stained against the mitochondria-specific cytochrome C and imaged (Fig. 6A). After imaging of an individual section was completed, the remaining fluorophores were photobleached at high laser intensities. In a second round, the sections were stained against mGFP and re-imaged (Fig. 6B). Because we did not observe any mGFP-positive signal within the postsynaptic cell (mGFP expression is strictly limited to the calyx), we are confident that fluorophores used in the first round of antibody labeling were photobleached completely and not transferred into a stable off-state. From these two data-sets, we were able to reconstruct a two-color super-resolution image (Fig. 6C). Dual color *tomo*STORM demonstrates a confinement of structures such as mitochondria within membranes (Fig. 6D) and fine structures of mitochondria can be readily appreciated (Fig. 6E).

We consider it important to validate super-resolution imaging data in several regards. First, electron microscopy data clearly illustrates that super-resolution microscopy requires tissue preparations maintaining excellent structural preservation (see Fig. 2). This is intuitively evident for studies aiming at localizing proteins at a resolution of ∼30 nm. Disrupted tissue, as encountered when using LRWhite resin, will not reveal interpretable localization and colocalization data. Second, cells and tissues with known ultrastructural features can be used to establish super-resolution approaches and aid in the interpretation of super-resolution images. For example, discontinuous distribution of mGFP could be established as a meaningful pattern because the shape of the calyx membrane was known. Third, immunostainings using an imaging approach with single molecule resolution require even more careful optimizations than in traditional approaches, simply because every single unspecific binding event will be detected (see Figs. 4,5). Using confocal microscopy, such signals would be invisible due to spatial averaging effects. Taken together, *tomo*STORM is capable of providing a nanoscopic representation of tissue architecture.

## Discussion

To understand cellular processes such as synaptic transmission, it is crucial to know the molecular composition and geometrical distribution of key molecular players on the nanometer scale in three dimensions. Various super-resolution microscopy techniques can provide excellent three-dimensional resolution [3], [18], [19]. Single-molecule methods which apply stochastic photoswitching are particularly promising, combining super-resolution imaging with the detection of single fluorophores and thus providing access to direct quantification. Single-molecule super-resolution imaging in 3D has been demonstrated in various approaches, e.g. using astigmatism [20], helical point-spread functions [21], interferometry [5] or dual-objective configurations [22]. Recently, 3D single-molecule super-resolution imaging was demonstrated at large sections of cultured neurons [23].

The essential idea behind *tomo*STORM is to combine the advantages of single-molecule based super-resolution imaging with the ones of array tomography, i.e. a large field of view easily accommodating several cells, an axial resolution determined by section thickness, multiplex-labeling and structural preservation. Using *tomo*STORM, we show for the first time super-resolution images of intact mammalian tissue at a resolution of 28 nm×28 nm×40 nm. This is three orders of magnitude higher than confocal microscopy, two orders of magnitude higher than the classic approach of array tomography [1], [24] and one order of magnitude higher than STED microscopy of serial thin sections [7]. In addition, we use bright and photostable fluorophores which provide a higher localization accuracy as e.g. fluorescent proteins which were used on single thin sections previously [6], and which were recently demonstrated in parallel imaging of six colors [25]. However, we note that both approaches can be readily combined [26] and offer benefits from orthogonal labeling strategies. Furthermore, protein tags that specifically bind small substrates labeled with organic fluorophores can be used [27].

Because our approach is capable of generating hundreds of sections [1], [24], there is no fundamental limit to cover thicker structures in 3D. At present, the time for imaging and data analysis is the limiting factor. Automated procedures of image acquisition and analysis might be a possible solution, such that the nanoscale distribution of proteins in entire cells and small tissue volumes can be routinely pursued. We provided proof of principle that multiple proteins can be localized simultaneously using *tomo*STORM. Repetitive cycles of staining and destaining or bleaching will make it possible to pursue nano-toponomics: identifying the positioning and geometric arrangement of large sets of proteins. This cannot be achieved with electron microscopy and will prove to be elementary to understand the contribution of nanoscale architecture to cellular function, which so far, has been the domain of models and indirect experimental inferences [28], [29].

## Materials and Methods

### Sample Preparation

All experiments were conducted in accordance with the German animal welfare guidelines and approved by the responsible authority (Regierungspräsidium Karlsruhe, Germany). All necessary steps were taken to ameliorate suffering of the rats, including systemic anesthesia (isofluorane) and local wound anesthesia (lidocaine), post-surgery pain treatment, professional handling, behavioural observation and housing in individually ventilated cages at 21°C and 55% humidity. The stereotaxic injections into the ventral cochlear nucleus (VCN) of two Sprague Dawley rats and tissue processing were done as reported previously [16], but using isoflurane inhalation anesthesia [30]. The production of rAAV encoding mGFP was done as reported previously [10]. Briefly, the VCN of rats was injected with 1 µl virus solution at P 3. After *in vivo* protein expression for the time period of 8 days rats were transcardially perfused with 20 ml of isotonic PBS followed immediately with 50 ml of 4% para formaldehyde in phosphate buffer 0.1 M pH 7.4. The brain was directly removed and stored over night at 4°C in the same fixative. 100 µm sections were cut on a Leica VT1400 vibratome through the MNTB region and further processed as described below.

### Resin Embedding

Resin embedding with LRWhite was done as described by Micheva et al. [1]. Briefly, the fixed tissue was further immersion fixed at 4°C overnight. After rinsing with PBS the tissue was dehydrated in a graded series of ethanol until complete dehydration with 100% ethanol. The tissue was then infiltrated with LRWhite resin (three times, 5 mins at 4°C and left overnight), embedded in gelatin capsules and polymerized at 50°C.

For Lowicryl HM20, tissue sections were high pressure frozen as described [31]. Freeze substitution was performed in a mixture of 3% uranyl acetate and methanol at −85°C for 70 h, followed by 3 washes in methanol at −85°C after which sections were warmed upto −40°C at 5°C/h. Tissue sections were infiltrated and embedded in Lowicry HM20 at −40°C and UV polymerized for 36 h at −40°C after which temperature was raised to room temperature. UV polymerization was continued for another 24 h.

### Ribbon Making

Ultrathin sections (40 nm) were cut with an ultramicrotome (Ultracut E, Reichert Jung, NY) using the procedure described by Harris et al. [32]. The ribbons obtained were mounted on coverslips which were made hydrophilic by treatment with a mixture of 1∶1 sulphuric acid and hydrogen peroxide. The ribbons were then further processed for immunostaining.

### Immunostaining

Following mounting on coverslips, the ribbons were washed in PBS (5 min) and immunostained following established protocols [33]. This was followed by blocking with 5% FCS in PBS. Primary antibodies were diluted in 5% FCS in PBS and applied on the ribbon for 2 hours. Primary antibodies used were anti-GFP (Cat# ab6556, Abcam) in 1∶200 dilution, and anti-cytochrome c oxidase (Cat# C9616, Sigma, US). Samples were washed 3 times with PBS. The appropriate secondary antibody diluted in 5% FCS in PBS was applied for 30 min followed by washing in PBS for 3 times. Secondary antibodies conjugated with Alexa Fluor 647 were purchased from Invitrogen (anti-rabbit (# A21245), anti-sheep (# A21448) and were used in the dilution 1∶200 (for anti rabbit) and 1∶1000 (for anti sheep). Secondary antibodies were custom-labeled with ATTO520 by conjugating the NHS ester of the fluorophore (Attotec, Germany) to unlabelled anti-rabbit antibody (#ab6016, Abcam) following the manufacturer's protocol. This antibody was used at a dilution of 1∶200.

### Electron microscopy

Electron microscopy was done following procedures described previously [16], but using 70 nm sections and no additional contrasting agents. Electron micrographs were taken with a Leo 906 E microscope.

### *d*STORM Imaging

*d*STORM images were recorded on a custom-built microscope using experimental protocols that were described earlier [13]. Briefly, a multi-line laser (Innova 70C, Coherent, USA) was coupled into an inverted microscope (IX71, Olympus, Japan), and the fluorescence signal was detected using an electron-multiplying charge-coupled device (EMCCD) (Ixon, Andor, Ireland) and appropriate filters and dichroic mirrors (AHF, Tübingen, Germany). Alexa Fluor 647 was photoswitched in oxygen-free aqueous buffer containing 100 mM mercaptoethylamine and using two illumination wavelengths, 488 nm (0.1–1 kW/cm^2^) for activation and 647 nm (1–5 k W/cm^2^) for read-out. Image reconstruction was performed using the rapidSTORM software package [34]. Typically, 8000 frames were recorded to reconstruct a *d*STORM image.

Dual-color *d*STORM imaging was performed by sequentially imaging Alexa Fluor 647. As a first target, cytochrome c oxidase was labeled, imaged and bleached. Successful bleaching was verified in control experiments to exclude that Alexa Fluor 647 was driven into long-lived dark states and recovered to fluorescence. In a next round, the ribbon was stained for anti GFP with Alexa 647 and imaged. For alignment of the *d*STORM images, we recorded wide field images of mGFP distribution in each of these steps. To achieve this in the first step, mGFP was stained and visualized with ATTO520.

### Image Reconstruction

The localization files containing the final super-resolved images were aligned using the MultiStackReg plugin of ImageJ [35]. The MultiStackReg plugin works by performing cross-correlations between pairs of adjacent images in the stack. It applies a transformation to one image and adjusts the parameters of the transformation to maximize the cross-correlation between the two adjacent images. To achieve dual color super resolution imaging, staining of the membrane-bound GFP (mGFP) was carried out using both Alexa Fluor 647 (used for *d*STORM) and ATTO520 (standard resolution image used as reference image for registering multiple channels). By way of reference images, registration between two distinct (cytochrome c and mGFP) super-resolution channels could be achieved. The aligned and surface rendered image stacks were visualized in 3D using Amira 4.1.2 software (Visage Imaging, Richmond, Victoria, Australia) (see also Figure S1).

## Supporting Information

Movie S1 Calyces of Held expressing mGFP. 53 consecutive sections imaged with dSTORM covering a volume of ∼50 µm×40 µm×2 µm.
(MOV)

Figure S1 Image registration procedure for one-color imaging. In a first step (i), the sum-TIRF images of individual sections were registered using MultiStackReg [35], producing a transformation matrix for consecutive sections. In a second step (ii), the transformation matrix was applied to register the corresponding super-resolution images of consecutive sections. (B) Image registration procedure for two-color imaging. As a reference image, a cellular structure was recorded in a spectrally separate channel for each section and prior to super-resolution imaging (i). The super-resolution image of the same section was recorded on another spectral channel, and after the recording, the fluorophores were photobleached. To image a second structure, the sample was re-stained, and the series of sections was recorded following the same procedure (ii). Registration of each super-resolution stack was performed using the transformation matrix obtained from registering the reference images recorded in each round of imaging (iii). As the same reference structure was used for each recording of a stack of super-resolution images, the different stacks could be overlaid.
(TIF)
