# Supplementary figures and images for: Three-Dimensional, Tomographic Super-Resolution Fluorescence Imaging of Serially Sectioned Thick Samples
Source: PLoS One. 2012 May 25;7(5):e38098. doi: 10.1371/journal.pone.0038098 (PMC3360663; doi:10.1371/journal.pone.0038098)

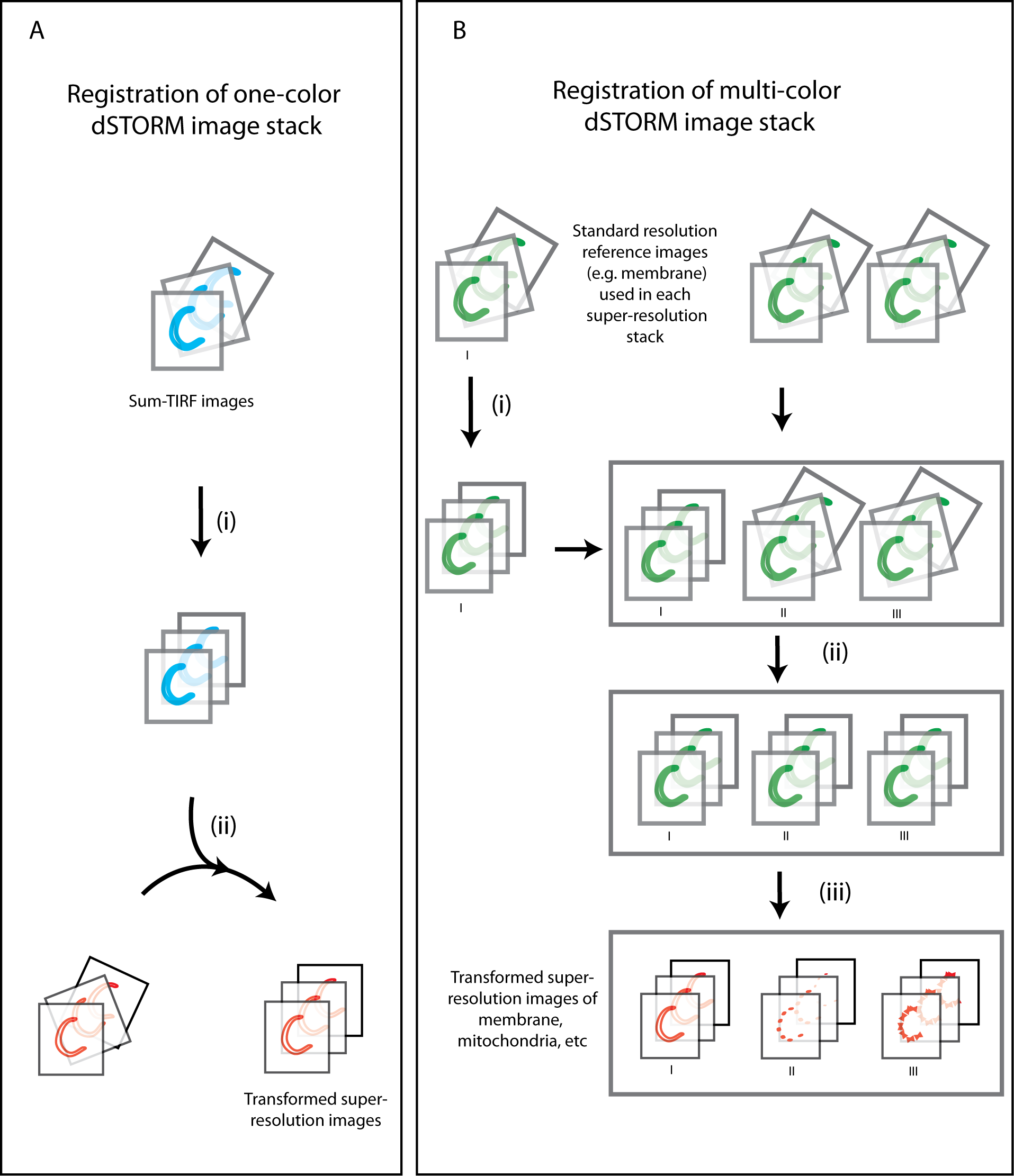

Supplement: Figure S1 — Image registration procedure for one-color imaging. In a first step (i), the sum-TIRF images of individual sections were registered using MultiStackReg [35], producing a transformation matrix for consecutive sections. In a second step (ii), the transformation matrix was applied to register the corresponding super-resolution images of consecutive sections. (B) Image registration procedure for two-color imaging. As a reference image, a cellular structure was recorded in a spectrally separate channel for each section and prior to super-resolution imaging (i). The super-resolution image of the same section was recorded on another spectral channel, and after the recording, the fluorophores were photobleached. To image a second structure, the sample was re-stained, and the series of sections was recorded following the same procedure (ii). Registration of each super-resolution stack was performed using the transformation matrix obtained from registering the reference images recorded in each round of imaging (iii). As the same reference structure was used for each recording of a stack of super-resolution images, the different stacks could be overlaid. (TIF) [file pone.0038098.s002.tif]
